# Cell-Based Therapies for Trabecular Meshwork Regeneration to Treat Glaucoma

**DOI:** 10.3390/biom11091258

**Published:** 2021-08-24

**Authors:** Shayshadri Mallick, Malini Sharma, Ajay Kumar, Yiqin Du

**Affiliations:** 1 Department of Ophthalmology, University of Pittsburgh, Pittsburgh, PA 15213, USA; SKM59@pitt.edu (S.M.); mas997@pitt.edu (M.S.); ajk157@pitt.edu (A.K.); 2 Department of Developmental Biology, University of Pittsburgh, Pittsburgh, PA 15213, USA; 3 McGowan Institute for Regenerative Medicine, University of Pittsburgh, Pittsburgh, PA 15213, USA

**Keywords:** glaucoma, trabecular meshwork, stem cells, regeneration, intraocular pressure

## Abstract

Glaucoma is clinically characterized by elevated intraocular pressure (IOP) that leads to retinal ganglion cell (RGC) and optic nerve damage, and eventually blindness if left untreated. Even in normal pressure glaucoma patients, a reduction of IOP is currently the only effective way to prevent blindness, by either increasing aqueous humor outflow or decreasing aqueous humor production. The trabecular meshwork (TM) and the adjacent Schlemm’s canal inner wall play a key role in regulating IOP by providing resistance when aqueous humor drains through the tissue. TM dysfunction seen in glaucoma, through reduced cellularity, abnormal extracellular matrix accumulation, and increased stiffness, contributes to elevated IOP, but current therapies do not target the TM tissue. Stem cell transplantation for regeneration and re-functionalization of damaged TM has shown promise in providing a more direct and effective therapy for glaucoma. In this review, we describe the use of different types of stem cells for TM regeneration in glaucoma models, the mechanisms of regeneration, and the potential for glaucoma treatment using autologous stem cell transplantation.

## 1. Introduction

Glaucoma is one of the leading causes of vision loss and complete blindness in the world today especially in African and Western countries affecting over 60 million people [1]. There are two main types of glaucoma, angle-closure and open-angle glaucoma. Angle-closure glaucoma occurs when the iris folds backward and bulges out to block the drainage of aqueous humor from the anterior chamber through the conventional outflow pathway, mainly the trabecular meshwork (TM) and the Schlemm’s canal, resulting in increased intraocular pressure (IOP) [2]. The more common type of glaucoma in African and Western countries, primary open-angle glaucoma (POAG), is caused due to reduced TM cellularity and an abnormal extracellular matrix (ECM) deposition in the TM [3,4]. The cells in the TM primarily function to balance the ECM turnover, phagocytose the debris through the outflow pathway, while aqueous humor drains through it to maintain normal IOP. Although the true disease pathogenesis is unclear, POAG has been associated with aging, family history, and genetics, as well as other risk factors such as hypertension, diabetes, and myopia [5]. In POAG, elevated IOP is a major risk factor for retinal ganglion cell (RGC) death, which relays visual information to the brain from the eyes via the optic nerve. The RGC death eventually leads to progressive vision loss [6]. Usually, by the time vision loss is noticeable, the RGC death is too far progressed, and the optic nerve has started to deteriorate which is essentially irreversible due to the optic nerve being incapable of regeneration, resulting in total blindness. IOP is the most treatable factor in POAG and thus has long been the target for reducing the eventual blindness that occurs from POAG. This reduction of IOP is currently achieved via a variety of ways, including drug therapies and surgical interventions. Rho-kinase inhibitors [7] can increase the contraction of cells in the TM. Adenosine agonists can modulate the outflow resistance of aqueous humor. Statins are repressors of SPARC (secreted protein acidic rich in cysteine, a fibrosis-related protein) found in TM cells. Beta-blockers function by reducing the production of aqueous humor [8,9]. Surgical procedures are also used, such as laser trabeculoplasty which applies laser energy to the TM network to increase drainage of aqueous humor, or incisional surgery which provides another route for aqueous humor egress [10]. However, these treatments do not always work with poor patient compliance and in most cases are not always permanent.

Since glaucoma is a degenerative disease, where TM and RGC cellularity is decreased [11,12], using stem cells to regenerate tissue is a promising area of study to explore more efficient and long-term treatment for glaucoma. Stem cells are immature cells that have not yet committed to a particular lineage of differentiation [13]. They are an appealing tool because they can divide in an immature state, and they can be induced to differentiate into many types of mature and functional cells. Stem cells can be classified based on their ability to differentiate into different cell types. Pluripotent stem cells, like embryonic stem cells and induced pluripotent stem cells (iPSCs), can differentiate into all cell types theoretically [14,15]. Multipotent stem cells are found in different tissues of the adult body (i.e., bone marrow and fat tissue) and are farther along the path of differentiation. Unlike pluripotent cells, multipotent cells have already committed to some particular lineages, so they have a more limited set of possible cell fates. Transplantation of stem cells, both pluripotent and multipotent, or differentiated functional cells from stem cells, in degenerative diseases is an encouraging area of study because of their regenerative and malleable characteristics. In glaucoma, pluripotent and multipotent stem cells have been studied in the context of both RGC and TM regeneration [16,17]. In this review, we will focus on the use of TM stem cells in TM regeneration, and touch on the use of other types of stem cells in TM regeneration for glaucoma.

## 2. Cell-Based Therapies for Trabecular Meshwork Regeneration by Using Trabecular Meshwork Stem Cells (TMSCs)

**Figure 1:**
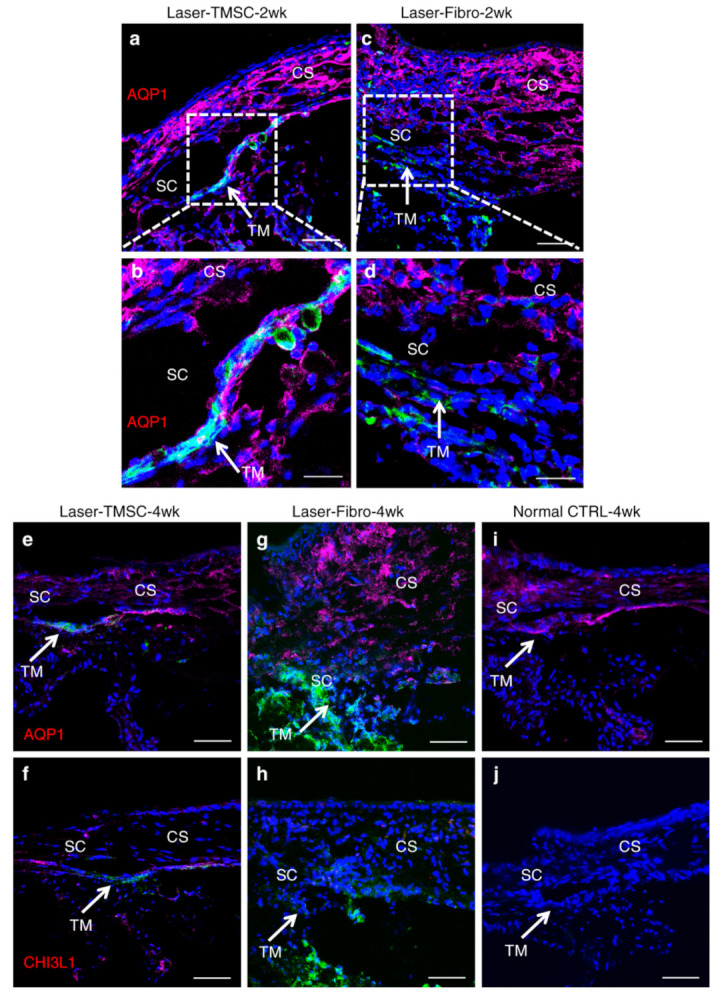
Transplanted TMSCs integrate into the host trabecular meshwork tissue. TMSCs integrated into all layers of the host trabecular meshwork tissue expressing AQP1 (**red**) as indicated by white arrows at 2 weeks after transplantation (**a**,**b**). Fibroblasts did not integrate into the TM and did not express AQP1 (**c**,**d**). At 4 weeks after transplantation, TMSCs integrated into the TM and expressed AQP1 (**e**) and CHI3L1 (**f**). Fibroblasts did not express AQP1 (**g**) or CHI3L1 (**h**). In non-treated mouse tissue, trabecular meshwork cells, endothelial cells, and keratocytes expressed AQP1 (**i**), whereas none of these mouse cells expressed CHI3L1 (**j**) (**note:** anti-CHI3L1 is a human-specific antibody and anti-AQP1 antibody recognizes both human and mouse antigens). Arrows point to the TM. Green indicates DiO-labeled (**injected**) cells. Scale bars, 50 μm (**a**,**c**,**e**–**j**); 20 μm (**b**,**d**). TM trabecular meshwork, SC Schlemm’s canal, CS corneal stroma. Adapted from Yun, H., et al. Commun Biol 1, 216 (2018) under the Creative Commons Attribution 4.0 International (CCBY4.0) license.

Various cell-based therapies for TM regeneration in glaucoma treatment have been proposed. The first and seemingly most effective one has been the TMSCs. These stem cells are tissue-specific stem cells in the TM [17,18,19,20,21,22], that can differentiate into phagocytic TM cells in vitro [22]. Upon being transplanted into the mouse anterior chamber, human TMSCs appear to be able to maintain mouse IOP in the normal range, improve aqueous outflow, home to the TM region, and suppress the inflammatory response [23]. Transplanted TMSCs can reconstruct the TM structure and rescue mouse eyes with normal range IOP in mice that had been treated with laser photocoagulation, which created similar conditions to POAG [24], but with an inflammatory response [25]. Our group has previously compared the TM localization and homing-in of TMSCs to human corneal stromal fibroblasts and a sham control in the healing of TM tissue wounds from laser photocoagulation [24]. TMSCs were seen to localize to the TM, specifically to the areas that received laser photocoagulation, while the fibroblasts seemed to localize non-specifically to the TM, the iris, and other tissues in the eye (Figure 1). The eyes injected with TMSCs also showed that there was very low-level apoptosis, as determined by terminal deoxynucleotidyl transferase-mediated dUTP-fluorescein nick end labeling (TUNEL) in the TM area, while the Sham and Fibroblast conditions showed a considerably greater level of apoptosis. TMSCs were also effective in reducing the inflammatory response induced by laser, as evaluated by the expression of the inflammatory markers CD45, CD11b, and F4/80, and also reducing fibrosis as assessed by the low expression of fibrotic markers SPARC and fibronectin (FN) in the eyes with laser photocoagulation [24]. On the contrary, the animals with sham and fibroblast injection were detected with higher inflammation and increased expression levels of SPARC and FN. Utilizing transmission electron microscopy (TEM), the structure of TM can be visualized. After 4 weeks of TMSC injection, in comparison to the sham and fibroblast conditions, there was a comparable number of organized cell-covered beams in the TM and giant vacuoles, like that of the control eyes, indicating structural and functional restoration of the TM [24].

The major parameter that has shown TMSCs to be a possible viable treatment for POAG is the ability for TMSCs to improve the outflow facility, maintain normal IOP, and prevent RGC loss [26]. In the laser photocoagulation-damaged mouse model, the TMSC treatment group had significantly reduced IOP in comparison to the fibroblast and sham groups [24]. In addition to this, the aqueous outflow facility, as measured following procedures from Lei et al. [27], was also shown to be increased after TMSC treatment as compared to fibroblast and sham treatment groups. This decrease in IOP and increase in outflow facility shows that the TMSCs can induce significant regeneration in glaucomatous eyes. Another study on a mouse POAG model with transgenic myocilin Y437H mutation (Tg-MyocY437H) [28] showed similar results [18]. In this study, human TMSCs were injected into the anterior chamber of 4-month old Tg-MyocY437H mice when they had elevated IOP. The mouse IOP was significantly reduced within a month after TMSC transplantation, eventually reduced to a value similar to that of wild-type (WT) mice [18]. Accompanying the IOP reduction, the outflow facility of the eyes was significantly increased to the level of WT mice. To achieve the treatment purpose for preventing vision loss, the mouse RGC were protected from death and the RGC function was preserved by measuring and comparing the mouse pattern electroretinogram (PERG) [18]. These studies, although done in mouse models, indicate that TMSC transplantation can be a promising approach to reduce IOP, increase outflow facility, and prevent vision loss for glaucoma treatment.

## 3. Cell-Based Therapies for Trabecular Meshwork Regeneration by Other Stem Cell Types

As described above, the use of stem cells for TM regeneration is a promising therapy for glaucoma treatment. TMSCs were shown to be able to home to the TM region specifically, differentiate into functional TM cells to increase the TM cellularity and improve the ECM components, and reduce IOP in mouses models of glaucoma [18,24]. Although TMSCs can suppress inflammatory response without evoking immunorejection after xenotransplantation [18,24], some patients may prefer to use cells from themselves. However, the use of autologous TMSCs can have some limitations. Firstly, TMSCs are found in the insert region of the TM [17,19,21], and harvesting enough of these cells from a living patient is difficult. Secondly, it is possible that there are fewer TMSCs in glaucoma patients, or that TMSCs isolated from these patients may be impaired or with genetic mutations. Because of these limitations, there has also been extensive research performed on the use of other types of stem cells for TM regeneration.

One of these cell types is induced pluripotent stem cells (iPSCs). iPSCs are adult somatic cells that have been genetically reprogrammed to express four pluripotent transcription factors Oct4, Sox2, Klf4, and cMyc [14] or OCT4, SOX2, NANOG, LIN28 [15]. This creates a system in which almost any cell type of the body can be derived from patient-specific iPSCs, using a variety of co-culturing techniques, mixtures of supplements, bioactive small molecules, and growth factors to control cell fate. Successful differentiation of both mouse and human iPSCs into TM-like cells has been carried out in multiple ways [29,30,31,32]. One method for TM regeneration and reduction of IOP in mouse models can be achieved using iPSCs that can be differentiated into TM cells (iPSC-TM). iPSC-TM are morphologically like primary TM cells that express proteins similar to that of the regular TM cells found in human eyes [8]. To form glaucomatous iPSC-TM, iPSCs must first be derived from fibroblasts isolated from transgenic mice that exhibit the glaucoma phenotype expressing human myocilin Y437H (Tg-MYOCY437H). These iPSCs can be induced to differentiate into TM cells using conditioned media (CM) by primary TM cells. Another method described co-culturing of mouse dermal fibroblast-derived iPSCs with primary human TM cells for 21 days [30]. By this time, the iPSC-TM cells share many characteristics with primary TM cells, including the ability to phagocytose particles and upregulation of myocilin and MMP3 in response to dexamethasone treatment. They also show reduced expression of pluripotency markers Nanog, Oct4, and Sox2, which is important in assessing if these cells can give rise to a tumor in vivo. This co-culture method of iPSC-TM induction was also successful when human iPSCs derived from dermal fibroblasts and keratinocytes were cultured with human primary TM cells [29]. We described another method of creating iPSC-TM cells from human iPSCs following a two-step induction process, where iPSCs are first induced into neural crest cells (NCC) and then TM-like cells [31]. This more closely mimics the path of differentiation for TM cells in embryonic development [33]. First, iPSCs are grown on an extracellular matrix (ECM) derived from the cell line A549 in N2B27 and Y27632 containing medium [31]. This has been shown to induce iPSCs into NCCs, as they began expressing NCC markers NGFR and HNK1 and reduced expression of the pluripotent stem cell marker SSEA4. These iPSC-NC cells were then grown on an ECM derived from cultured primary TM cells and in primary TM conditioned media (CM) for 10 to 14 days. After this culture protocol, the iPSC-TM cells shared many characteristics with primary TM cells, including increased expression of the TM cell maker CHI3L1, and increased expression of myocilin, in response to five days of 100 nM dexamethasone treatment. After 14 days of dexamethasone treatment, these iPSC-TM cells formed cross-linked actin networks (CLANs), a structure predominately formed in TM cells after dexamethasone treatment. This method of iPSC-TM induction breaks up the protocol into two parts, allowing expansion and storage of iPSC-NC cells from which to derive iPSC-TM cells in the future. It also describes a method that relies on isolated ECM and CM from primary TM cells instead of direct co-culture.

Regardless of the method of iPSC-TM induction, these cells have successfully rescued glaucoma phenotypes in a transgenic mouse model of glaucoma [34,35]. As briefly described above, the transgenic-MYOCY437H mice express human myocilin with the disease-causing mutation Y437H. This mutation prevents myocilin from being transported out of the endoplasmic reticulum (ER), thus causing ER stress [28]. In one study, mouse-derived iPSCs were differentiated into iPSC-TM cells through co-culture with TM cells [34]. After a 14-day induction in the CM yielded cells that exhibited morphology and gene expression similar to that of primary TM cells. Specifically, a marked expression of laminin A4 and tissue inhibitor of matrix proteases 3 (TIMP3). iPSC-TM cells were then isolated and transplanted through intracameral injections into four-month-old Tg-MYOCY437H and WT mice. This was compared to mice injected with either PBS (vehicle control) or an equal number of fibroblasts. Six weeks after injection, the vehicle control Tg-MYOCY437H mice showed an increase in IOP, and a decrease in aqueous humor outflow compared to WT mice, confirming that the transgenic mice displayed a glaucoma phenotype. However, when Tg-MYOCY437H mice were transplanted with iPSC-TM cells, IOP decreased and aqueous humor outflow increased, matching WT levels. This observation was held for the remainder of the study (nine weeks). Twelve weeks after injection, mice were assessed for iPSC-TM integration and RGC preservation. Compared to the vehicle control, glaucoma mice injected with iPSC-TM cells showed both an increased RGC and TM cell density, similar to the levels seen in WT mice. Though injected iPSC-TM cells were able to integrate into the TM tissue, the increased TM cells in the host TM were not iPSC-TM cells, suggesting that injected cells may have induced proliferation of endogenous TM cells. To look at this observation in vitro, this group transfected the MYOCY437 mutant into primary mouse TM cells and co-cultured them directly and indirectly (using cell inserts) with iPSC-TM cells. They found that co-culture with iPSC-TM cells promoted TM cell proliferation, but only when these cells were in direct contact with each other. This study demonstrated that in the transgenic MYOCY437H mouse model, iPSC-TM injection rescued the glaucoma phenotype with decreasing IOP, increasing aqueous humor outflow, and maintaining RGC density by promoting the endogenous proliferation of TM cells. Similar effects were also observed in aged mice when transplantation of iPSC-TM cells was done at six months of age, instead of four months [35].

Human-derived iPSC-TM cells have also been used in TM regeneration of older human eyes maintained in perfusion culture [29]. Like in glaucomatous eyes, TM cellularity also decreases with age. For each pair of donated eyes, iPSC-TM were created from donor-specific fibroblasts and perfused into one eye, while the other was maintained as a non-injected control. At both 7 and 14 days, the eye perfused with iPSC-TM cells maintained normal IOP and showed increased proliferation of endogenous TM cells compared to the control eye. Like the previous study, in vitro co-culture of human iPSC-TM with the TM5 cell line promoted TM5 cell proliferation only when these cells were in contact with each other. This suggests that the proliferative effect of iPSC-TM cells on TM cells is not mediated through paracrine factors. However, the iPSC-TM transplanted eyes showed no increase in outflow facility as compared to the control eyes, indicating that the iPSC-TM only increase the cellularity of the TM and had no real effect in ameliorating POAG. The observed results might also be possible because the eyes used in the experiment initially did not have an elevated IOP as well as not being able to survive the perfusion organ culture (POC) procedure for more than 3 weeks, resulting in errors in the experiment. The results may have differed if the study was repeated with eyes that contained the POAG phenotype results. Though this study did not use donors with glaucoma, the effects they observed held true for most donors, suggesting that the effect of the iPSC-TM transplant would be similar among patients with different genetic backgrounds. The use of iPSC-TM cells in TM regeneration of glaucomatous eyes shows promise, but the exact mechanism of how iPSC-TM cells can promote proliferation of the endogenous TM through direct cell contact, and how this translates to rescue of a glaucoma phenotype, remains unclear.

Another type of stem cell that has been studied within the context of TM regeneration are the adipose-derived stem cells (ADSCs). Like iPSCs, ADSCs can be obtained for autologous transplantation, but they do not require genetic perturbation. Additionally, large numbers of ADSCs can be isolated using minimally invasive procedures and can be differentiated into many cell types, making them another promising candidate for tissue regeneration. The method of TM regeneration using ADSCs begins like that of iPSCs, where ADSCs are differentiated into TM-like cells in vitro [36]. We have previously shown that ADSCs can be successfully differentiated in TM cells using TM-ECM and conditioned media. The ADSC-TM cells thus obtained showed increased expression of TM cell markers CHI3L1 and AQP1 [37]. In our recent study [36], ADSCs were isolated from three donors and subsequently cultured with either TM cells, ECM and CM obtained from TM cells, or only ECM from TM cells. After 10 days of culture, ADSCs in the first two groups began expressing TM markers CHI3L1 and AQP1, and reduced expression of stem cell marker OCT4 [36]. They also exhibited phenotypic traits of TM cells, including increased expression of myocilin and increased CLAN formation in response to dexamethasone treatment. ADSC-TM cells also showed more phagocytic activity compared to uninduced ADSCs. Interestingly, ADSCs incubated with TM-ECM only did not show obvious characteristics of the above-mentioned, implicating the importance of TM paracrine factors in this differentiation. Undifferentiated ADSCs and ADSC-TM from the ECM+CM group were then isolated and injected into wild-type (WT) mice, along with a fibroblast control. One month later, ADSCs and ADSC-TM cells integrated into the TM tissue, although some ADSCs were off target, while fibroblasts showed more off-target attachment to the iris and corneal endothelium, similar to previous reports [23,24]. Both ADSCs and ADSC-TM cells expressed the TM cell marker AQP1, which modulates aqueous outflow, while fibroblasts did not. Transplants of ADSCs and ADSC-TM maintained normal IOP and aqueous humor outflow levels in WT mice, while fibroblast injection increased IOP and reduced the aqueous humor outflow facility. This study describes another promising method for stem cell treatment of glaucoma, as ADSCs can be isolated in large quantitates with minimally invasive techniques and can be induced into ADSC-TM cells. After transplant into WT mouse anterior chambers, they preferentially integrate into the TM and maintain normal IOP and aqueous humor outflow. We have shown that ADSC conditioned media can induce regeneration in TM cells by increasing their wound healing potential and reducing fibrosis in vitro [37]. It would be interesting to see the effect of ADSC and ADSC-TM transplants specifically in a mouse model of glaucoma.

In addition to ADSCs, mesenchymal stem cells (MSCs) have also been used in TM regeneration [38,39]. MSCs are a multipotent stem cell population found in many adult tissues including the bone marrow. They have been studied for the treatment of many degenerative diseases because, in addition to their ability to differentiate into many cell types [40,41,42,43]. They also have been shown to secrete a variety of biomolecules that promote endogenous cell growth [44,45]. A rat model of glaucoma used a laser to damage the anterior chamber angle of the eye, causing increased IOP that persisted for thirty days [38]. In this study, injection of MSCs significantly reduced IOP compared to injections of saline or fibroblasts. After thirty days, histology analysis of the TM showed intact structures for MSC injected rats, while rats without MSC transplant showed increased TM scarring. Even though the injected cells did not remain integrated into the TM past ninety-six hours, twenty-four hours after injection, researchers observed that the MSCs preferentially integrated into the damaged tissue. However, co-culture of MSCs and TM cells showed that the MSCs did not exhibit any TM-like phenotypes, suggesting the MSCs did not differentiate into TM cells in vivo. Instead, this implicated the role of paracrine factors in the reduction of the IOP observed, as IOP in MSC injected rats remained low for thirty days, well after the injected MSCs were cleared. To assess these possible effects, 40x concentrated CM isolated from MSCs grown in normal vs. hypoxic conditions were injected into laser-treated rats. These experiments showed that injection of MSCs and injection of CM from hypoxic MSCs both increased aqueous humor outflow and decreased IOP, while saline, fibroblasts, and CM from normal MSCs had no effect. Taken together, these results suggest that paracrine factors from MSCs under stress can mediate TM tissue regeneration after laser treatment. MSCs and MSCs-CM were able to decrease IOP, increase aqueous humor outflow, restore TM structure, and promote preferential integration of progenitor cells into newly damaged TM. We observed similar effects in our recent study where paracrine factors (secretome) from TMSC were found to reduce IOP significantly as compared to sham (basal medium) and secretome derived from fibroblasts in steroid-induced and primary open-angle glaucoma Tg-MyocY437H mouse models [46]. TMSC secretome also rescued TM and RGC death in both animal models. In addition, the TMSC secretome also reversed the glaucomatous effect of dexamethasone on TM cells by reducing glaucoma markers (Myocilin and ANGPTL7) and restoring TM functional markers (CHI3L1 and AQP1). Another study with MSCs used a slightly different glaucoma model in rats [39]. IOP was elevated by the cauterization of three episcleral veins in these rat eyes. Injection of MSCs successfully reduced IOP in these animals for 13 days and preserved RGC density compared to mice injected with media only. They also observed that MSCs integrated into the TM for 24 days after injection. In vitro, this study also showed that a culture of TM cells with MSC-CM improved TM cell viability and eased TM contraction. Though somewhat different from each other, these studies were able to show that MSC transplant into different glaucoma models reduced IOP and preserved TM structure, not by differentiating into TM-like cells, but most likely through secretion of paracrine factors. Another in-depth study investigated the expression and phenotype differences between MSCs and TM cells to further distinguish the two and described a way to accurately define a functional TM cell [47]. The use of MSCs is different from the use of iPSCs and ADSCs, as MSCs are not induced into TM cells before transplantation. The use of stem cell paracrine factors also opens the possibility of a cell-free therapy in the treatment of glaucoma, distinct from the reported iPSC-TM transplant, which seems to require direct contact between exogenous and endogenous cells to induce a beneficial effect. Table 1 describes important studies undertaken in the past using different types of stem cells for glaucoma treatment.

## 4. Mechanisms of Cell Mediated Glaucoma Treatment

The main mechanisms involved in the development of POAG is yet to be properly deduced. However, there are a few hypotheses about what may result in the development of POAG. One of these is the interaction between the chemokine SDF1 and its receptor CXCR4, which has been shown to have an important role in hematopoietic stem cell homing or localization [48]. The CXCR4 receptor has also been implicated in the signaling and homing of the cells involved in lymphopoiesis, myelopoiesis, embryogenesis, angiogenesis, cardiogenesis, neuron migration, and cerebral development [49,50]. Due to its important function in all these processes, it could play a role in TMSC homing to TM tissue to repair any damage and/or induce proliferation. Gene expression studies of TMSCs and TM cells did indicate that there was a high expression of CXCR4, indicating a possibility that it was involved in TMSC signaling and localization. To confirm this interaction, Yun et al. cultured TMSCs on a TM feeder treated with recombinant SDF1α and 1β and anti-SDF 1 antibody to neutralize SDF1 in the TM cells [24]. The use of the anti-SDF 1 antibody neutralized the expression of SDF1, while the TMSCs cultured with the SDF1αβ displayed an increase in SDF1 expression. It was observed that the greatest number of TMSCs attached to the TM-SDF1αβ cells, while the least TMSCs attached to TM-SDF1Ab, indicating an increased affinity of TMSCs to the TM cells with upregulated SDF1. To inhibit the CXCR4 axis, TMSCs were treated with the inhibitor IT1t [51]. After the inhibitor treatment, TMSCs were not able to attach to TM and showed no significant difference in their affinity towards TM cells. It was also observed that chemotaxis between TMSCs and TM cells without direct contact utilized untreated TM cells, TM cells with SDF1αβ, and TM cells with SDF1Ab. A larger percentage of the TMSCs were found to migrate to the TM cells treated with SDF1αβ while showing a reduced migration towards TM cells treated with SDF1Ab. Further, the use of a CXCR4 antagonist, AMD3100, which reduced CXCR4 expression in TMSCs, and a short hairpin RNA which reduced SDF1 expression in TM, showed a decrease in the attraction between the TM and TMSCs, indicating that the CXCR4/SDF1 chemokine axis is part of the signaling involved in the TMSC homing-in and migration to the TM cells. This cell homing is essential to glaucoma treatment, as it ensures that the TMSCs can localize to the TM tissue to properly rebuild the TM network and reduce the IOP associated with POAG, to prevent any further RGC death and vision loss.

Another mechanism that is important in the TMSC-mediated protection of the TM tissue, as well as the maintenance of the TM Extracellular matrix (ECM) and protection of RGCs, is through the TMSCs upregulating certain genes related to these functions. Using transcriptomic analysis, Xiong et al. showed the involvement of three upregulated pathways in TMSCs that showed increased TM ECM interaction which included the focal adhesion pathway, the PI3K-Akt signaling pathway, and the ECM-receptor interaction pathway [18]. The focal adhesion pathway proteins are especially important as adhesion receptors for the ECM as well as involved in the signaling downstream for processes such as apoptosis, contraction, endocytosis, and phagocytosis [52,53]. The PI3K-Akt signaling pathway usually responds to oxidative stress in the regular TM cells as it is heavily involved with recovering from abnormal morphological changes and can cause cytoskeletal changes in the TM [34]. The ECM-receptor pathway has been shown to control the ECM of the TM cells which control the amount of outflow resistance that would in turn have an effect on controlling the IOP. These pathways were downregulated in the fibroblasts in comparison to TMSCs. This might be a reason that TMSCs were able to promote a regenerative effect on the damaged TM and reduced risk factors leading to POAG while the fibroblasts showed no such effect.

Our recent study showed that α5β1 Integrin promotes the homing-in potential and anchorage of the TMSCs to the damaged TM. TMSCs express α5β1 Integrin at higher levels as compared to corneal fibroblasts [53]. The α5β1 Integrin anchor helps TMSCs to attach to damaged TM cells with an upregulated fibronectin. Blocking the α5β1 Integrin by using an antibody resulted in the abolishment of the capacity of TMSCs to attach to the TM tissue, causing non-retention of TMSCs in TM cells after one month of injection.

Another mechanism that has been proposed to treat POAG and alleviate the symptoms and risk factors associated with it, has been increasing the number of endogenous cells in the TM. In their experiment, Zhu et al. showed that after iPSC-TM transplantation, there was a reduction in the IOP and increase in outflow facility in Tg-MYOCY437H mice in comparison to the animals which received fibroblasts, and a vehicle control [34]. The authors observed an increase in TM cellularity and a decrease in the other risk factors associated with POAG. To test that it was the implanted iPSC-TM cells that gave rise to these new TM cells since the transgenic animals expressed the cellular marker dsRed using histochemical evaluation, the number of dsRed cells could be counted and recorded. Upon counting these cells in the eye that received the iPSC-TM implantation, it was noted that the number of dsRed positive cells was relatively low even though the number of TM cells and the TM cell density had increased significantly. This indicated that the increased TM cellularity was increased as a result of endogenous TM cell proliferation and not because of the iPSC-TM cells that were implanted. Thus, the transplantation of these iPSC-TM cells helped the endogenous TM cells to proliferate and increase the density of the existing TM already. To confirm this, Zhu et al. utilized an in vitro experiment which involved introducing untreated primary TM cells to media alone, TM cells with adenoviral vectors expressing the Y437H myocilin mutation (Ad5RSV-myocilinY437H) alone, and TM cells with Ad5RSV-myocilinY437H and iPSC-TM cells. The cells with Ad5RSV-myocilinY437H alone showed a slightly lower growth than the control cells as expected due to the diseased myocilin phenotype, and the cells that were grown in contact with the iPSC-TM cells showed significantly higher growth in comparison to the TM cells alone. The experiment was then performed again with the primary TM cells physically separated from the iPSC-TM, and it was seen that the growth was comparable to the control cells. Along with this, when TM cells were in direct contact with the iPSC-TM cells, it was noted through the Panther Classification System [54] that the genes being expressed in the endogenous TM cells that received iPSC-TM cells were related to cellular processes, especially the cell cycle. This confirmed that the exogenous iPSC-TM cells were driving the proliferation of the endogenous TM cells and increased the cellularity, leading to the TM regeneration and IOP reduction seen in the mouse eyes. However, this required direct contact between the TM cells and the iPSC-TM cells for this increased endogenous cell proliferation. Further studies are needed to elucidate why endogenous TM cell proliferation in the Tg-MYCY437H mice can promote TM regeneration and reduce IOP since the TM cells are still abnormal myocilin mutant cells.

**Figure 2:**
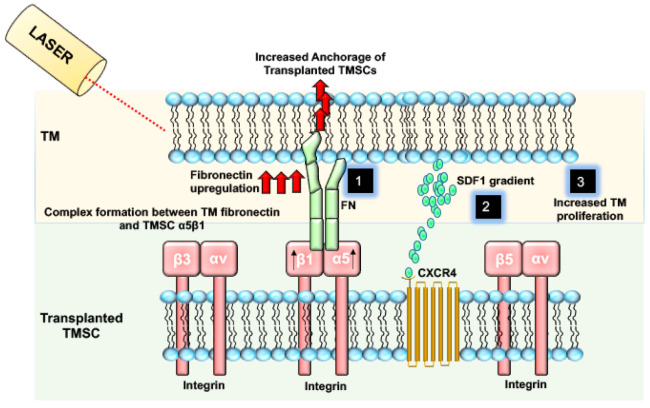
Transplanted TMSCs integrate into the host trabecular meshwork via various mechanisms to induce regeneration. Post Laser damage to TM results in increased FN. TMSC with high expression of α5β1 Integrin can anchor to the damaged TM by forming a complex between α5β1 Integrin and FN (1). TMSCs also contain CXCR4 and can bind to TM cells with an increased expression and gradient of SDF-1 (2). TMSCs can also induce the proliferation of TM cells in the TM tissue, resulting in the maintenance of TM homeostasis and reduced IOP (3).

Figure 2 describes various mechanisms by which stem cells home in and induce regeneration in glaucoma.

## 5. Conclusions

Stem cell transplantation for TM regeneration remains an important area for exploring new effective glaucoma treatment options. Many different types of stem cells can be used in TM regeneration, and it seems they work in similar, but distinct ways. Not only is the process of stem cell isolation specific to each stem cell type, but the exact mechanism of TM regeneration may also be distinct. TMSCs home and integrate into the TM tissue, naturally differentiate into functional TM cells, and secrete factors that promote tissue regeneration. iPSCs are genetically modified and induced into TM-like cells before transplant. After transplant, they do not further differentiate, and instead promote proliferation of endogenous TM cells, mediated through direct cell-cell contact. ADSCs can be transplanted without induction or can be induced to differentiate into TM-like cells before transplant. MSCs are not induced into TM-like cells before transplantation and do not differentiate into functional TM cells post-transplant. Instead, it seems that paracrine factors secreted by MSCs under stress conditions promote endogenous TM re-functionalization. All these studies resulted in reduced IOP and increased TM cellularity, highlighting the numerous possibilities for glaucoma treatment using various types of stem cells. It is necessary to further investigate the mechanisms of how each type of stem cells distinctly promotes these beneficial effects, but these studies represent a promising avenue for glaucoma treatment.

## Figures and Tables

**Table 1:** Studies involving the use of different stem cells for glaucoma treatment.

Study	Cell Type	Origin	Model	Route of Transplantation	Main Findings	Reference
Use stem cells to treat Tg-MYOCY437H POAG mouse model	TMSC	Human	Mouse Transgenic Age 4–6 months	Intracameral	IOP normalization, increased TM cellularity and outflow facility, TM regeneration, reduction of ER stress, modulated ECM, restored ultrastructure of TM tissue, rescued RGC number and function	[18]
Use stem cells in C57BL/6 WT mice	TMSC	Human	Mouse C57BL/6 WT (adult 10 weeks)	Intracameral	IOP normalization, TMSC homing to the TM, TMSC function like TM in vivo, no immunorejection with xenotransplant	[23]
Use stem cells in laser photocoagulation damaged mice	TMSC	Human	Mouse C57BL/6 WT (Adult 10 weeks)	Intracameral	IOP normalization, outflow facility normalization, TMSC home to damaged TM region, TMSC function like TM in vivo, suppressing inflammation	[24]
Use stem cells to treat Tg-MYOCY437H POAG mouse model	iPSC	Mouse	Mouse Transgenic Age 4 months	Intracameral	IOP normalization, increased outflow facility, increased endogenous TM cellularity, rescue of RGC number.	[34]
Use stem cells to treat Tg-MYOCY437H POAG mouse model	iPSC	Mouse	Mouse Transgenic Age 6 months	Intracameral	IOP normalization, increased outflow facility, increased endogenous TM cellularity, restore TM structure, preservation of ER structure.	[35]
Use stem cells in ex vivo human ocular perfusion organ culture system	iPSC	Human	Ex vivo human eyes Age 79.2 yrs ± 14.6	ex vivo perfusion	IOP normalization, increased endogenous TM cellularity, outflow facility normalization	[29]
Use stem cells in ex vivo human ocular perfusion organ culture system	iPSC	Human	Ex vivo human eyes using saponine to damage the TM cells	ex vivo perfusion	Restoring TM cellularity and IOP homeostatic function after iPSC-TM perfusion	[32]
Use stem cells in C57BL/6 WT mice	ADSC	Human	Mouse C57BL/6 WT (Adult 10 weeks)	Intracameral	IOP normalization, outflow facility normalization, ADSC-TM and ADSCs home to TM, ADSC-TM and ADSCs function like TM in vivo (increased AQP1 expression).	[36]
Use stem cells in laser-damaged ocular hypertension rat model	MSC	Mouse	Rat with laser damage to half circumference of anterior chamber angle	Intraocular	IOP normalization, increase outflow facility, rescue of RGC number, restore structure of TM tissue. May be through paracrine factors.	[38]
Use stem cells in vessel cauterized ocular hypertension rat model	MSC	Rat	Rat Long Evans WT with 3 cauterized episcleral veins	Intracameral	IOP normalization, rescue of RGC number, rescue TM cell viability, restore TM structure. May be through paracrine factors	[39]

Abbreviations: TMSC—Trabecular Meshwork Stem Cells, POAG—Primary Open-Angle Glaucoma, IOP—Intraocular Pressure, ER—Endoplasmic Reticulum, RGC—Retinal Ganglion Cells, ECM—Extracellular Matrix.
